# Main right hepatic duct entering the cystic duct: a case report

**DOI:** 10.1186/s40792-019-0604-y

**Published:** 2019-03-25

**Authors:** Toshiya Abe, Shinichiro Ito, Yoshikazu Kaneda, Ryuichiro Suto, Shinji Noshima

**Affiliations:** Department of Surgery, Yamaguchi Prefectural Grand Medical Center, Yamaguchi, Japan

**Keywords:** Aberrant bile duct, Bile duct injury, Laparoscopic cholecystectomy

## Abstract

**Background:**

Risk factors for bile duct injury in laparoscopic cholecystectomy include severe inflammation at Calot’s triangle and aberrant bile duct variations. Knowledge of the various biliary anomalies and early identification may therefore assist in decreasing the rate of bile duct injury.

**Case presentation:**

A 65-year-old woman was admitted with right hypochondrial pain and high fever. A diagnosis of acute calculous cholecystitis was made by radiological imaging. Magnetic resonance cholangiopancreatography revealed that the confluence of the right and left hepatic duct was unclear. Intraoperatively, the procedure was converted from a laparoscopic cholecystectomy to laparotomy because of unclear anatomy of the cystic duct with severe inflammation at Calot’s triangle. Furthermore, intraoperative cholangiography from Hartmann’s pouch showed the main right hepatic duct entering the cystic duct. Subtotal cholecystectomy was performed to avoid injuring the right hepatic duct.

**Conclusion:**

Although an aberrant hepatic duct entering the cystic duct is not uncommon, the main right hepatic duct infiltrating the cystic duct is extremely rare. Preoperative and intraoperative evaluation of the biliary duct and awareness of aberrant biliary duct variations is important in preventing bile duct injury.

## Background

Bile duct injury in laparoscopic cholecystectomy (LC) can cause bile leakage and stenosis. Risk factors for bile duct injury in LC include severe inflammation at Calot’s triangle and aberrant bile duct variations [1]. Knowledge of the various biliary anomalies and early identification may therefore assist in decreasing the rate of bile duct injury. We report an extremely rare case of the main right hepatic duct entering the cystic duct and discuss the importance of the preoperative and intraoperative evaluation of the biliary duct.

## Case presentation

**Fig. 1:**
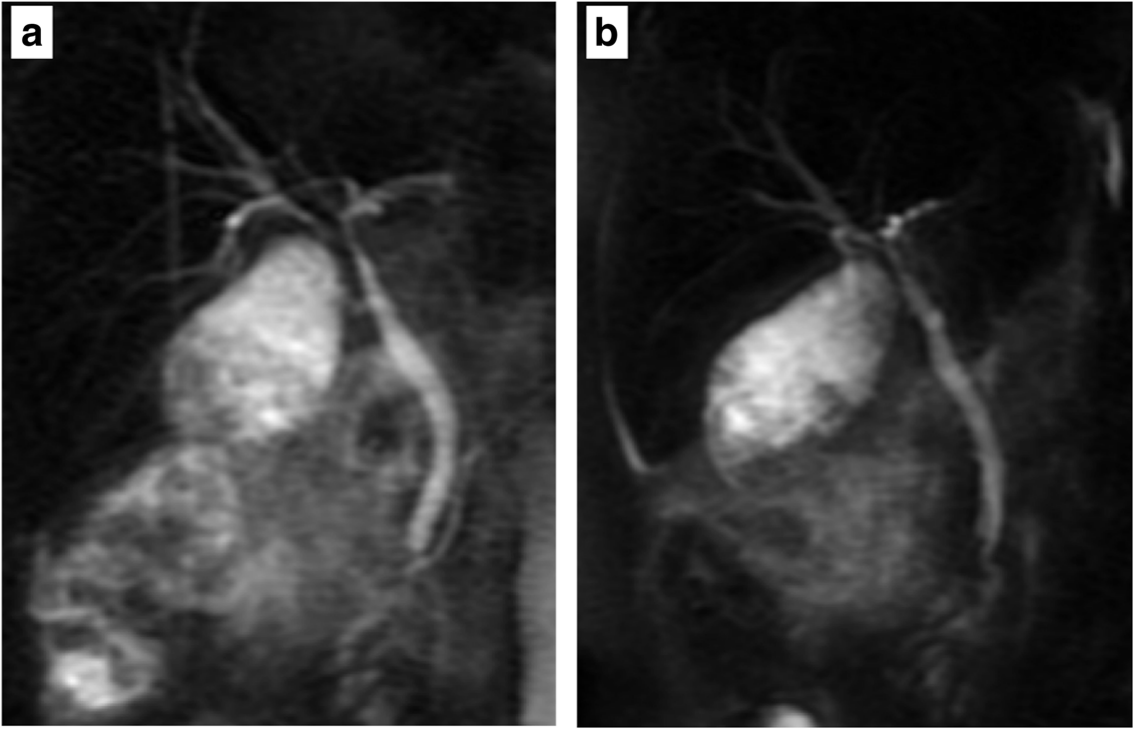
**a** Magnetic resonance cholangiopancreatography (MRCP) showed that the confluence of the right and left hepatic duct was unclear. **b** MRCP (right anterior oblique view)

**Fig. 2:**
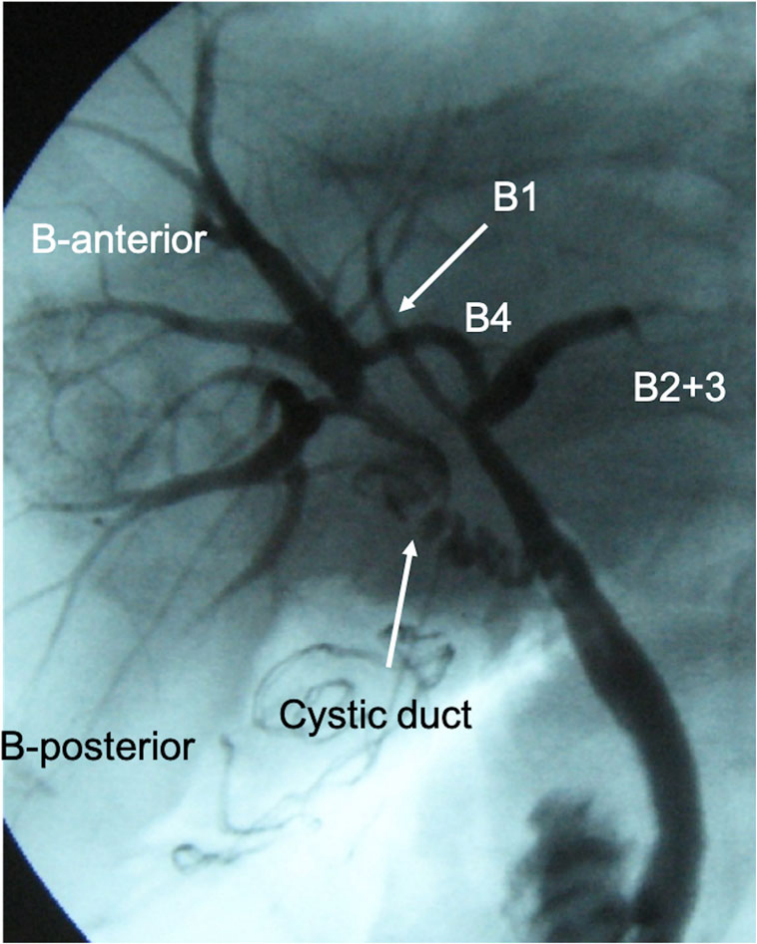
Intraoperative cholangiography revealed the main right hepatic duct entering the cystic duct

**Fig. 3:**
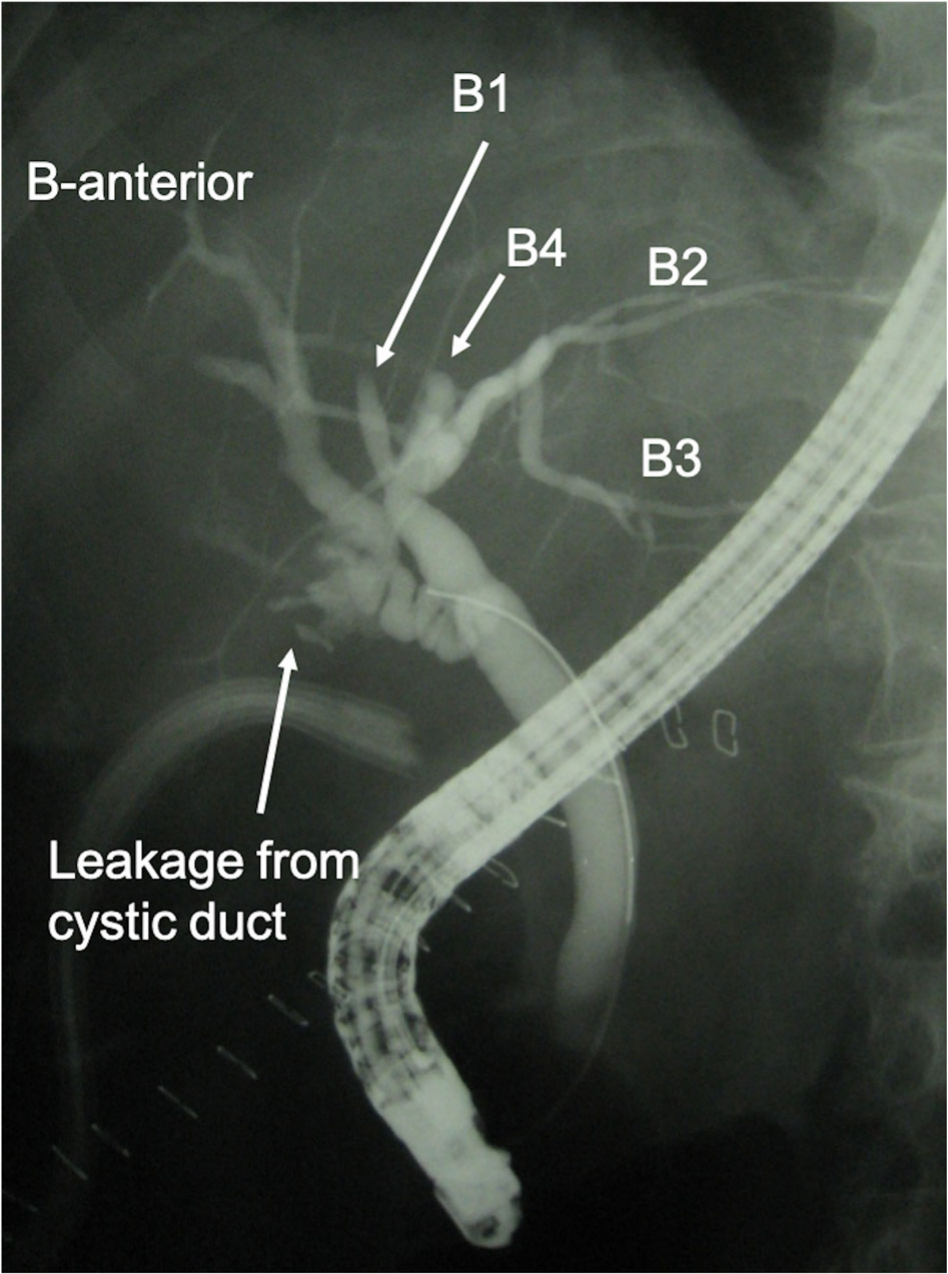
Postoperative cholangiography by endoscopic retrograde cholangiopancreatography revealed the main right hepatic duct entering the cystic duct and bile leakage from the stump of the gallbladder neck

A 65-year-old woman was admitted with right hypochondrial pain and high fever. On physical examination, her vital signs were as follows: temperature, 38.0 °C; blood pressure, 140/82 mmHg; heart rate, 80 beats per minute and regular; respiratory rate, 14/min; and peripheral capillary oxygen saturation, 98% at room air, respectively. Her consciousness was lucid and Murphy’s sign was positive. Laboratory evaluation showed an increase in inflammatory response with a white blood cell count of 23,200/mm and C-reactive protein level of 30.5 mg/dL without liver, renal, and hematological dysfunction. Abdominal ultrasonography revealed acute calculous cholecystitis with thickened wall and gallbladder stone. Magnetic resonance cholangiopancreatography (MRCP) showed that the confluence of the right and left hepatic duct was unclear (Fig. 1). Under preoperative diagnosis of acute calculous cholecystitis (grade II) [2], an emergency LC was planned. However, the procedure was converted to open cholecystectomy (OC) because of unclear anatomy of the cystic duct with severe inflammation at Calot’s triangle. Because identification of the cystic duct was difficult, the gallbladder was incised at the level of Hartmann’s pouch, and intraoperative cholangiography (IOC) from Hartmann’s pouch showed the main right hepatic duct entering the cystic duct (Fig. 2). After identifying the aberrant bile duct, subtotal cholecystectomy was performed to avoid injuring the aberrant hepatic duct. The gallbladder neck was closed by suture and ligation without approaching the cystic duct. A postoperative complication of bile leakage from the resection stump of the gallbladder was treated by endoscopic nasobiliary drainage (Fig. 3). The patient was discharged on postoperative day 25.

## Discussion

The incidence of bile duct injury, one of the most severe complications of LC, has been reported to be about 0.5% [3]. Risk factors for bile duct injury in LC include severe inflammation at Calot’s triangle and aberrant bile duct variations [1]. Previous reports revealed that the frequency of bile duct injury in cases with aberrant bile duct was higher than in cases without it [4]. Although the occurrence of an aberrant hepatic duct entering the cystic duct is not uncommon [5, 6], the main hepatic duct infiltrating the cystic duct is extremely rare. In the past literature, we found only ten reports of the main hepatic duct entering the cystic duct, including the present case [5–12] (Table 1). In this type of the aberrant bile duct, the ligation of the cystic duct in proximity to the junction of the right hepatic duct may cause severe complications. A previous report showed that ligation of the main hepatic duct may lead to functional loss of a large segment of liver, jaundice, and cholangitis [13]. Therefore, if this anomaly has been identified, we must attempt to prevent injury to the main hepatic duct by resecting the distal cystic duct of the junction at the hepatic duct.

**Table 1:** Previous reports of the main right hepatic duct entering the cystic duct

Author	Year	Age	Sex	Diagnosis of aberrant right hepatic duct	Operation
Prinz	1976	52	F	IOC	OC
Puente	1983	Unknown	Unknown	IOC	Unknown
Champetier	1991	Unknown	Unknown	IOC	Unknown
Champetier	1991	Unknown	Unknown	IOC	Unknown
Nomura	1999	31	M	PTGBD	LC
Hashimoto	2002	Unknown	Unknown	IOC	LC
Yamamoto	2003	41	F	IOC	LC
Kayahara	2005	69	F	ERCP	LC → OC
Matsumoto	2008	59	M	PTGBD, ERCP	LC
Present case	2018	65	F	IOC, ERCP	LC → OC

*IOC* intraoperative cholangiography, *OC* open cholecystectomy, *LC* laparoscopic cholecystectomy, *PTGBD* percutaneous transhepatic gallbladder drainage, *ERCP* endoscopic retrograde cholangiopancreatography

Previously, MRCP in this anomaly showed the absence of union of the right and left hepatic duct [10, 11]. Similar to previous reports, MRCP in our case also showed that the confluence of the right and left hepatic duct was unclear. However, it is difficult to precisely diagnose this aberrant bile duct solely by the lack of clarity at this junction. Therefore, keeping this rare anomaly in mind, we might consider utilizing another imaging modality, such as drip infusion cholecystocholangiography–computed tomography (DIC-CT) and IOC. Kurata et al. [14] reported that it was possible to identify an aberrant right posterior sectoral hepatic duct (PHD) by using DIC-CT when the presence of aberrant PHD was suspected but could not be confirmed by MRCP or endoscopic retrograde cholangiopancreatography (ERCP). Furthermore, Hirao et al. [15] reported that DIC-CT clearly identified both aberrant bile ducts and cystic ducts in comparison with MRCP. Therefore, it may be useful to perform DIC-CT when the presence of an aberrant bile duct is suspected by MRCP.

Tokyo Guidelines 2018 [2, 16] recommends early LC performed by experienced surgeons for grade II acute cholecystitis if patients meet the criteria of Charlson comorbidity index ≤ 5 [17] and American Society of Anesthesiologists physical status classification ≤ 2 [18]. Although the present case met these criteria of early LC, the procedure was converted from LC to OC because of the unclear anatomy of the cystic duct with severe inflammation at Calot’s triangle. Furthermore, because IOC from Hartmann’s pouch revealed this aberrant right hepatic duct, subtotal cholecystectomy was performed to avoid injuring it. Similar to our case, most previous cases have involved adequate diagnosis of the aberrant right hepatic duct by IOC. Although it remains controversial whether IOC can prevent iatrogenic bile duct injury during LC [3, 19], Törnqvist et al. [20] reported that IOC was associated with a reduced risk of bile duct injury especially in patients with concurrent, or a history of, acute cholecystitis. Therefore, IOC might be useful for the avoidance of bile duct injury when this type of anomaly is suspected by preoperative imaging in addition to the diagnosis of acute cholecystitis.

## Conclusion

We report an extremely rare case of the main right hepatic duct entering the cystic duct. Preoperative and intraoperative evaluation of the biliary duct and awareness of the variations of aberrant biliary duct are important in preventing bile duct injury in LC.

## Abbreviations

CT: Computed tomography
DIC-CT: Drip infusion cholecystocholangiography–computed tomography
ERCP: Endoscopic retrograde cholangiopancreatography
IOC: Intraoperative cholangiography
LC: Laparoscopic cholecystectomy
MRCP: Magnetic resonance cholangiopancreatography
OC: Open cholecystectomy
PHD: Posterior sectoral hepatic duct
